# Longitudinal Gut Microbiome Changes Associated with Transitions from *C. difficile* Negative to *C. difficile* Positive on Surveillance Tests

**DOI:** 10.3390/microorganisms13102277

**Published:** 2025-09-29

**Authors:** L. Silvia Munoz-Price, Samantha N. Atkinson, Vy Lam, Blake Buchan, Nathan Ledeboer, Nita H. Salzman, Amy Y. Pan

**Affiliations:** 1 Department of Medicine, Medical College of Wisconsin, 8701 Watertown Plank Road, Milwaukee, WI 53226, USA; 2 Center for Microbiome Research, Medical College of Wisconsin, 8701 Watertown Plank Road, Milwaukee, WI 53226, USA; saatkinson@mcw.edu (S.N.A.); nsalzman@mcw.edu (N.H.S.); 3 PITA Analytics, P.O. Box 96, Laupahoehoe, HI 96764, USA; 4 Department of Pathology, Medical College of Wisconsin, 8701 Watertown Plank Road, Milwaukee, WI 53226, USA; bbuchan@mcw.edu (B.B.); nledeboe@mcw.edu (N.L.); 5 Department of Pediatrics, Medical College of Wisconsin, 8701 Watertown Plank Road, Milwaukee, WI 53226, USA

**Keywords:** gut microbiome, *Clostridioides difficile*, *Clostridioides difficile* infection, microbiome composition, longitudinal

## Abstract

*Clostridioides difficile* is an obligate anaerobe and is primarily transmitted via the fecal–oral route. Data characterizing the microbiome changes accompanying transitions from non-colonized to *C. difficile* colonized subjects are currently lacking. In this retrospective cohort study, we examined 16S rRNA gene sequencing data in a total of 481 fecal samples belonging to 107 patients. Based on *C. difficile* status over time, patients were categorized as Negative-to-Positive, Negative Control, and Positive Control. A linear mixed effects model was fitted to investigate the changes in the Shannon α-diversity index over time. Zero-inflated negative binomial/Poisson mixed effects models or generalized linear mixed models with negative binomial/Poisson distribution were used to investigate the changes in taxon counts over time among different groups. A total of 107 patients were eligible for the study. The median number of stool samples per patient was 3 (IQR 2–4). A total of 42 patients transitioned from *C. difficile* negative to positive (Negative-to-Positive), 47 patients remained negative throughout their tests (Negative Control) and 18 were always *C. difficile* positive (Positive Control). A significant difference in microbiome composition between the last negative samples and the first positive samples were shown in Negative-to-Positive patients, ANOSIM *p* = 0.022. In Negative-to-Positive patients, the phylum Pseudomonadota and family *Enterobacteriaceae* increased significantly in the first positive samples compared to the last negative samples, *p* = 0.0075 and *p* = 0.0094, respectively. Within the first 21 days, Actinomycetota decreased significantly over time in the Positive Control group compared to the other two groups (*p* < 0.001) while Bacillota decreased in both the Negative-to-Positive group and Positive Control. These results demonstrate that the transition from *C. difficile* negative to *C. difficile* positive is associated with alterations in gut microbial communities and their compositional patterns over time. Moreover, these changes play an important role in both the emergence and intensification of the gut microbiome dysbiosis in patients who transitioned from *C. difficile* negative to positive and those who always tested positive.

## 1. Background

*Clostridioides difficile* is a Gram-positive, spore-forming, obligate anaerobe. *C. difficile* spores are resistant to alcohol-based cleaner; therefore, they are prevalent in hospital environments and asymptomatic carriers may contribute significantly to disease transmission [1]. Colonization does not always happen after spore exposure as a favorable gastrointestinal environment is also needed. Healthy people with diverse microbiota may react to initial spore exposure differently from individuals whose metabolic and microbial environment of the gut has been disturbed [2]. Toxins can be generated by colonization, which can further lead to toxin-mediated inflammation and disease [3]. The protein toxins, TcdA and TcdB, are two major virulence factors of *C. difficile* that are produced by most clinical isolates [4]. Hypervirulent strains, such as PCR ribotypes 027 and 078, commonly produce an additional toxin called *C. difficile* binary toxin (CDT), which contributes to increased virulence and disease severity by disrupting the host cell actin cytoskeleton and enhancing adherence to host cells [5,6,7].

*C. difficile* is primarily transmitted via the fecal–oral route and can cause toxin-mediated *C. difficile* infection (CDI). CDI is a major cause of antibiotic-associated diarrhea worldwide and the most common cause of healthcare-associated infections in the United States [8,9]. Known risk factors of developing CDI include exposure to antimicrobial agents, old age, medication with proton pump inhibitors, long-term hospitalization, gastrointestinal surgery or gastrointestinal procedures [10,11], some of which have been shown to be linked to gut microbiota dysbiosis [12]. The gastrointestinal microbial community and the host are crucial in disease development throughout the life cycle of *C. difficile*.

In a recent report, the Centers for Disease Control and Prevention (CDC) names *C. difficile* an urgent antibiotic resistance threat to the American population. In 2017, the CDC estimated 223,900 cases of CDI occurred in hospitalized patients with 12,800 attributable deaths [13]. Despite a decrease in hospital onset CDI [14], it still causes considerable morbidity and mortality in the U.S. population and thus, novel interventions aimed at decreasing the incidence of CDI are warranted. U.S. hospitals implement many preventative interventions to halt the occurrence of CDI among hospitalized patients. These interventions include environmental disinfection, hand hygiene, and antimicrobial stewardship. Even though the results are mixed, the use of probiotics or prebiotics for the prevention of CDI has been explored, especially for secondary prevention of CDI (i.e., recurrences). Modulation of the microbiome, such as the use of fecal microbiota transplantation, has been widely successful for the treatment of CDI and prevention of recurrences [15]. These findings, along with basic science research, highlight the relevance of gut microbiome regulation on CDI. *C. difficile* colonization is a necessary biological step for the development of CDI [16,17]; however, data characterizing the microbiome changes accompanying transitions from non-colonized to *C. difficile* colonized are currently lacking.

As part of a Quality Improvement initiative, our hospital has been systematically screening patients for the presence of *C. difficile* since 2016. Using this stool collection, we aimed to determine the microbiome characteristics of patients who transitioned from *C. difficile* negative to *C. difficile* positive using systematic stool surveillance tests. We hypothesize that patients who transitioned from *C. difficile* negative to *C. difficile* colonized on surveillance tests will have structural microbiome differences when compared to patients who remained *C. difficile* negative or those who tested persistently *C. difficile* positive on all surveillance tests.

## 2. Methods

### 2.1. Study Subjects

This retrospective cohort study was performed at a 607-bed, licensed teaching affiliated hospital in the Greater Milwaukee area. This hospital admits an annual average of 31,234 patients and has approximately 175,817 inpatient-days per year. As part of a quality improvement project, which started in late 2016, inpatient units with the highest *C. difficile* infection rates underwent *C. difficile* surveillance testing on admission and weekly thereafter. These units included two hematology–oncology wards (66 beds; 18,935 patient days/year), a solid organ transplant unit (27 beds; 7455 patient days/year), a general medical unit (32 beds; 8060 patient days/year), and the medical intensive care unit, MICU (20 beds; 7800 patient days/year). Surveillance tests were part of the admission orders in these selected units; samples were collected by nursing staff using a *C. difficile* surveillance order to distinguish them from specimens ordered by clinical teams based on symptoms and submitted to the clinical microbiology laboratory for processing (see below). Stool samples were kept refrigerated at −4 °C and promptly transferred to the research laboratory. In the research laboratory, all surveillance and clinical stool samples were catalogued and cryopreserved at −80 °C within a stool biorepository approved by the Medical College of Wisconsin’s Internal Review Board (IRB; PRO00031186). The present study was approved by MCW’s IRB with a waiver of informed consent (PRO00028305). Demographic data and clinical data were collected as detailed in Table 1.

This study included all consecutive patients tested at least twice for the presence of *C. difficile* with at least one of these tests being a surveillance test. Rectal or perianal swabs were not included. Results were categorized as (a) Negative-to-Positive (*C. difficile* negative on initial test(s) and positive in subsequent *C. difficile* test(s)), (b) Negative Control (all colonization tests and any clinical tests remained negative), or (c) Positive Control (patients who always tested *C. difficile* positive). Patients who tested initially *C. difficile* positive and subsequently tested negative were excluded. Also excluded were patients who fluctuated more than once from *C. difficile* negative to positive or vice versa.

### 2.2. Microbiology

Both *C. difficile* colonization tests and clinical tests (ordered by clinical teams based on symptoms) were performed using the Xpert *C. difficile* Assay (Cepheid, Sunnyvale, CA, USA). This test targets a conserved region of the cytotoxin B (*tcdB*) gene which is required for virulence. The test was independently validated internally for off-label use with formed stools. Formed stools were tested by inserting the swab into or rolling the swab on the surface of the stool specimen until the swab was visibly coated. The swab was then eluted into Xpert *C. difficile* test buffer (provided) and tested in accordance with the product insert. During the validation, the limit of detection (LoD) for alternative sources was assessed using swabs coated with a stool matrix that tested negative for *C. difficile* and subsequently submerged in 10-fold dilutions of a *C. difficile* (ATCC: BAA-1875) suspension in normal saline. The LoD was found to be approximately 5 × 10^3^ cfu/swab, or 5 × 10^4^ CFU/mL. This is 1 log_10_ greater than the LoD stated by the manufacturer.

Since March 2017, all positive *C. difficile* Nucleic Acid Amplification Test (NAAT) clinical tests (not including colonization tests) underwent reflex testing for TcdA and TcdB toxin using the *C. diff* Quik Chek Complete lateral flow test (Alere, Waltham, MA, USA). This test provides a result for glutamate dehydrogenase (GDH) and a combined result for TcdA/TcdB.

### 2.3. Stool Sample Processing

Stool samples underwent DNA extraction using the QIAamp Fast DNA Stool Mini Kit (Qiagen, Germantown, MD, USA). Purified genomic DNA was submitted to the University of Wisconsin-Madison Biotechnology Center for sequencing. DNA concentration was verified fluorometrically using either the Qubit^®^ dsDNA HS Assay Kit or Quant-iT™ PicoGreen^®^ dsDNA Assay Kit (ThermoFisher Scientific, Waltham, MA, USA). Samples were prepared in a similar process to that described in Illumina’s 16s Metagenomic Sequencing Library Preparation Protocol, Part # 15044223 Rev. B (Illumina Inc., San Diego, CA, USA) with the following modifications: The 16S rRNA gene V3/V4 variable region was amplified with fusion primers (forward primer 341f: 5′-ACACTCTTTCCCTACACGACGCTCTTCCGATCT(N)_0/6_CCTACGGGNGGCWGCAG-3′, reverse primer 805r: 5′-GTGACTGGAGTTCAGACGTGTGCTCTTCCGATCT(N)_0/6_GACTACHVGGGTATCTAATCC-3′). The quality and quantity of the finished libraries were assessed using an Agilent 4200 TapeStation DNA 1000 kit (Agilent Technologies, Santa Clara, CA, USA) and Qubit^®^ dsDNA HS Assay Kit (ThermoFisher Scientific, Waltham, MA, USA), respectively. Libraries were pooled in an equimolar fashion and appropriately diluted prior to sequencing. Paired end, 300 bp sequencing was performed using the Illumina MiSeq Sequencer and a MiSeq 600 bp (v3) sequencing cartridge. Images were analyzed using the standard Illumina Pipeline, version 1.8.2. Data were rarefied to control for uneven sequencing depth between samples.

### 2.4. Statistical Methods

Data were summarized as median and interquartile range (IQR) or *n* (%). A chi-square or Fisher’s exact test was used to compare categorical variables. A *t*-test or Analysis of Variance (ANOVA) was performed to compare continuous variables between groups. To satisfy parametric assumptions, we performed log transformations for some analyses. A non-parametric Mann−Whitney−Wilcoxon test or Kruskal−Wallis test was employed where parametric assumptions were not satisfied.

A linear mixed effects model was fitted to investigate the changes in the Shannon α-diversity index over time between different groups of patients. A zero-inflated negative binomial/Poisson mixed effects model or generalized linear mixed effects models with negative binomial/Poisson distribution were used to examine the changes in taxon counts over time between different groups [18]. Line plots were used to show the fitted models without adjustment. Nested models were compared using a likelihood ratio test. Due to the short follow-up time (median and IQR 4 (0, 8) days) in the Positive Control group, we only employed the first 21 days for analyses involved in this group. We also determined the changes in alpha diversity and taxon counts of the last negative samples and first positive samples in Negative-to-Positive patients using a paired t test or Wilcoxon signed-rank test.

Differences in microbiome community compositions (β-diversity) were compared using Principal Coordinates Analysis (PCoA) in conjunction with Permutational Multivariate Analysis of Variance (PERMANOVA) based on a Bray−Curtis distance matrix. The means of ranked dissimilarities were compared by the Analysis of Similarities (ANOSIM) test.

A *p* < 0.05 was considered significant. Statistical analyses were performed using SAS 9.4 (SAS Institute, Cary, NC, USA) and R 4.5.1 (R Core Team, Vienna, Austria).

## 3. Results

### 3.1. Study Population

A total of 107 patients were eligible for the study. Of these, 42 patients transitioned from *C. difficile* negative to positive (Negative-to-Positive), 47 patients remained negative throughout their tests (Negative Control) and 18 were always *C. difficile* positive (Positive Control). Table 1 depicts the demographics of all three groups. Overall, 55% were males with a median age of 65 years (IQR 54–71). Most patients (82%) were White with only 14% self-described as African American. A total of 46% were bone marrow transplant recipients and 18% were admitted to intensive care units. A high percentage (94%) of patients had exposure to antimicrobial medications, including antibiotics with activity against anaerobes (87%), and antifungals (75%). The median number of comorbidities was 6 (IQR 4–9). Statistically significant differences between the three groups (Table 1) were found around race (*p* = 0.019), ICU stay (*p* = 0.024), and antibiotic exposures (*p* = 0.010), including antibiotics with activity against anaerobes (*p* < 0.001) and antifungal exposures (*p* = 0.011).

### 3.2. Taxonomic Composition

**Figure 1:**
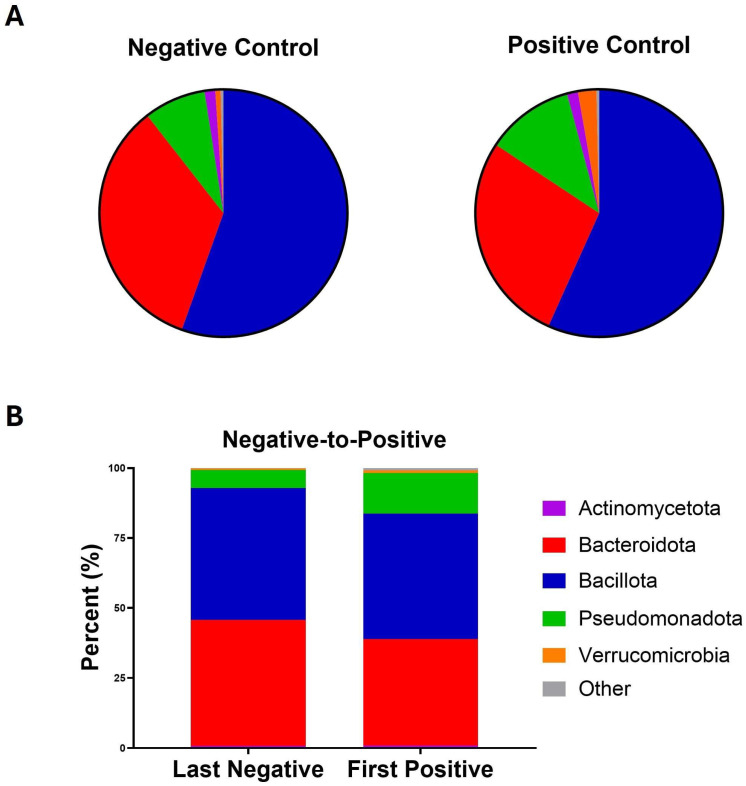
Bacterial composition at the phylum level among groups. (**A**) Pie chart showing the relative abundance of the most abundant phyla in Negative Controls (286 samples) and Positive Controls (41 samples). (**B**) Stacked bar plot showing the relative abundance of phyla in Negative-to-Positive patients’ last negative and first positive samples.

A total of 481 fecal samples were available for analysis. The median number of stool samples per patient was 3 (IQR 2–4). Out of the 481 stool samples, 278 (58%) were surveillance samples and 203 (42%) were stool samples ordered by clinical teams. There were 154 stool samples from Negative-to-Positive patients, 286 from Negative Controls and 41 from Positive Controls. Each sample had a median of 75 (IQR 47–103) discrete bacterial taxa. At the phylum level, Bacillota, Bacteroidota, Pseudomonadota, and Actinomycetota were 53.1%, 35.6%, 8.7%, and 1.3% of the total reads, respectively. In Negative-to-Positive patients, the last negative samples had 45.0% Bacteroidota, 47.2% Bacillota, and 6.4% Pseudomonadota while the first positive samples had 37.9%, 44.8%, and 14.5%, respectively (Figure 1B). Figure 1A shows the average relative abundance of dominant phyla in Negative Controls and Positive Controls and Supplementary Figure S1 displays these over time. *Bacteroidaceae* (31.0%), *Lachnospiraceae* (15.7%), *Enterococcaceae* (9.7%), *Oscillospiraceae* (8.3%), *Enterobacteriaceae* (6.7%), *Streptococcaceae* (5.5%), and *Lactobacillaceae* (5.0%) were dominant families contributing 5% or more of the total reads.

### 3.3. Diversity Changes

**Figure 2:**
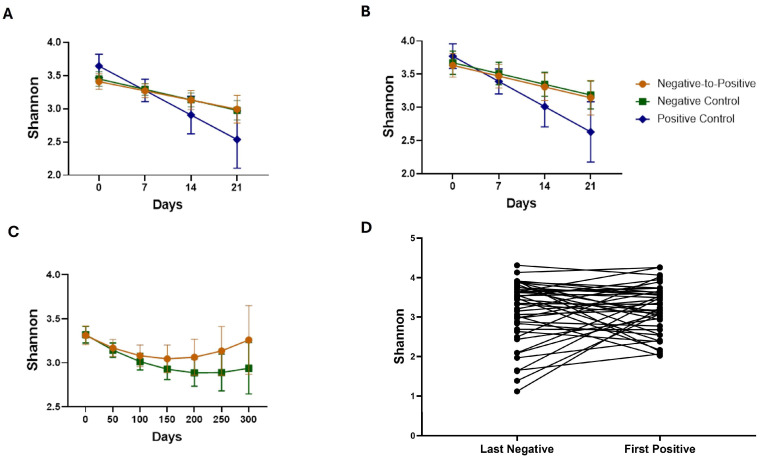
Shannon α-diversity index changes over time. (**A**) Line plot showing the model fitted estimates in the first 21 days among three groups without adjustment. (**B**) Line plot showing the model fitted estimates in the first 21 days among three groups adjusted for anerobic antibiotics. (**C**) Line plot showing the model fitted estimates using all time points in Negative-to-Positive group and Negative Control group. (**D**) Changes in last negative and first positive samples in Negative-to-Positive patients.

**Figure 3:**
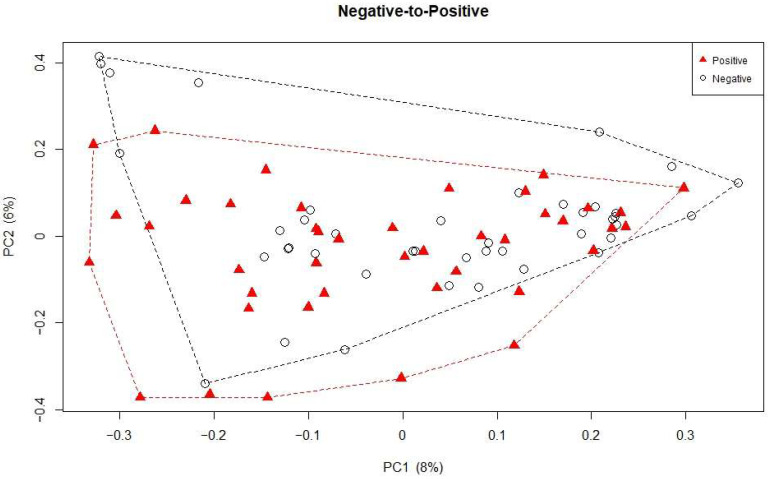
Beta diversity last negative vs. first positive. Principal Coordinates Analysis (PCoA) plot showing variation in beta diversity between the last negative samples and the first positive samples. Each point represents an individual patient. Dashed line represents a convex hull around the outermost points of each group.

As shown in Figure 2A, the Shannon index (an alpha-diversity metric) decreased over time in the first 21 days in Negative-to-Positive patients (slope = −0.20, *p* = 0.092), Negative Controls (slope = −0.023, *p* = 0.014), and Positive Controls (slope = −0.053, *p* = 0.027), but there were no significant differences between the three groups. The results were similar after adjusting for antifungals, aerobic antibiotics, or antibiotics with activity against anaerobes (Figure 2B). Of note, patients who received anerobic antibiotics showed a significant decrease in the Shannon index compared to those who did not, *p* = 0.015. No significant differences in the Shannon index were found between Negative-to-Positive patients and Negative Controls when samples from all time points were included (Figure 2C). Although no significant changes in the Shannon index in Negative-to-Positive patients were observed when patients transitioned from negative to positive (Figure 2D), the differences in microbiome composition were significant (Figure 3), ANOSIM *p* = 0.022.

### 3.4. Longitudinal Composition Changes

**Figure 4:**
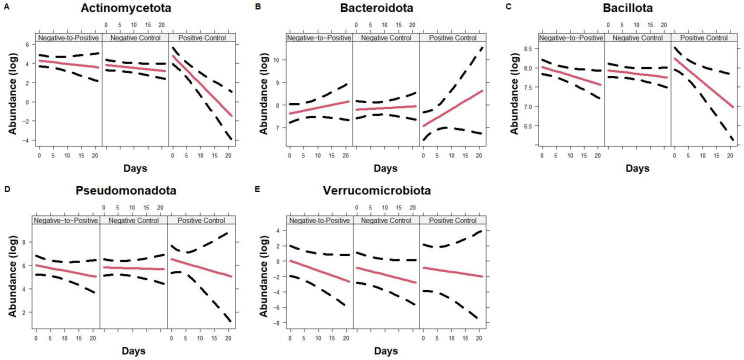
Trajectory of Phyla up to 21 days. Line plots showing phyla Actinomycetota (**A**), Bacteroidota (**B**), Bacillota (**C**), Pseudomonadota (**D**), and Verrucomicrobiota (**E**) changes over time in all groups. Lines represent the fitted model and the dotted line represents the 95% CIs. Data are presented in a natural log scale.

**Figure 5:**
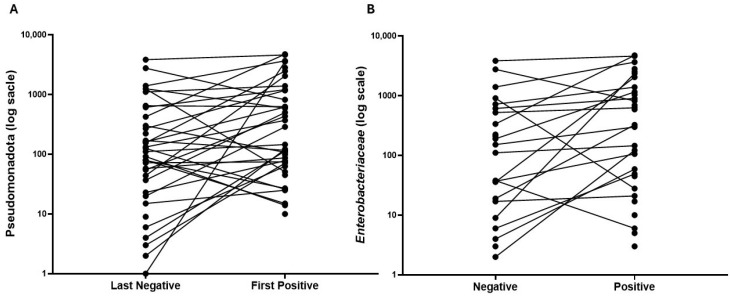
Changes of phylum and family in Negative-to-Positive group. Line plots showing the changes from last negative samples to first positive samples in phylum Pseudomonadota (**A**) and family Enterobacteriaceae (**B**).

Within the first 21 days, Actinomycetota did not differ at the baseline between the three groups but decreased significantly over time in the Positive Control group compared to the Negative-to-Positive group (*p* < 0.001) and the Negative Control group (*p* < 0.001) (Figure 4A). Bacillota decreased over time in the Negative-to-Positive group (*p* = 0.032) as well as the Positive Control group (*p* = 0.010), although baseline values and slopes were not significantly different between these two groups (Figure 4C). Patterns in Bacteroidota, Pseudomonadota, and Verrucomicrobiota were not different between the groups in the first 21 days. When all time points were included, baselines values and changes over time in Actinomycetota, Bacteroidota, Bacillota, Pseudomonadota, and Verrucomicrobiota did not differ between Negative Control and Negative-to-Positive groups (Supplementary Figure S2). Pseudomonadota increased significantly in the first positive samples when compared to the last negative samples in Negative-to-Positive patients, median and IQR count 76 (4, 271) vs. 134 (54, 1164), *p* = 0.0075 (Figure 5A).

**Figure 6:**
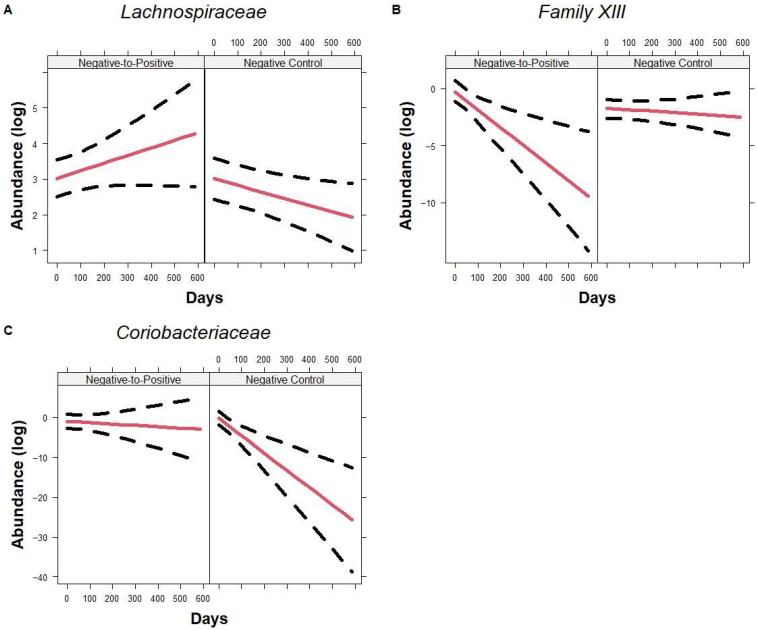
Trajectory of family using all time points. Line plots showing family *Lachnospiraceae* (**A**), *Family XIII* (**B**), and *Coriobacteriaceae* (**C**) changes over time in Negative-to-Positive group and Negative Control group. Lines represent the fitted model and the dotted line represents the 95% CIs. Data are presented in natural log scale.

There were no significant differences in baseline values or slopes at the family level between the three groups within the first 21 days. *Peptostreptococcaceae* (*p* = 0.019) and *Barnesiellaceae* (*p* = 0.035) decreased significantly while *Pseudomonadaceae* increased significantly (*p* = 0.0061) in the Negative-to-Positive group compared to the Positive Control group. When including all time points, *Family XIII* was higher at the baseline level in the Negative-to-Positive group but decreased significantly compared to the Negative Control group (Figure 6B), *p* = 0.0082 and 0.0016, respectively. In contrast, *Lachnospiraceae* (*p* = 0.0095) and *Coriobacteriaceae* (0.0032) decreased significantly over time in the Negative Control group compared to the Negative-to-Positive group although baseline values were similar (Figure 6A or Figure 6C). *Clostridiaceae 1* (*p* = 0.020) was higher at the baseline while *Streptococcaceae* (*p* < 0.001) and *Micrococcaceae* (*p* = 0.037) were lower in the Negative-to-Positive group when compared to the Negative Control group. Additionally, *Oscillospiraceae* (*p* = 0.032), *Prevotellaceae* (*p* = 0.017), *Acidaminococcaceae* (*p* = 0.019), and *Barnesiellaceae* (*p* = 0.016) decreased significantly over time in Negative-to-Positive patients.

**Figure 7:**
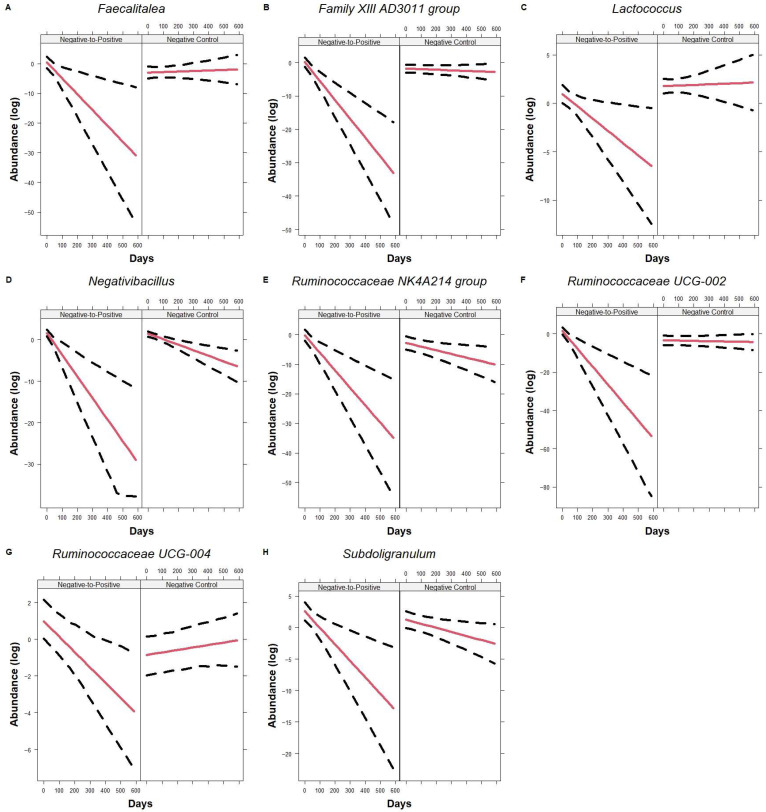
Trajectory of genus using all time points. Line plots showing genera *Faecalitalea* (**A**), *Family XIII AD3011 group* (**B**), *Lactococcus* (**C**), *Negativibacillus* (**D**), *Ruminococcaceae NK4A214 group* (**E**), *Ruminococcaceae UCG-002* (**F**), *Ruminococcaceae UCG-004* (**G**), and *Subdoligranulum* (**H**) changes over time in Negative-to-Positive group and Negative Control group. Lines represent the fitted model and the dotted line represents the 95% CIs. Data are presented in natural log scale.

**Figure 8:**
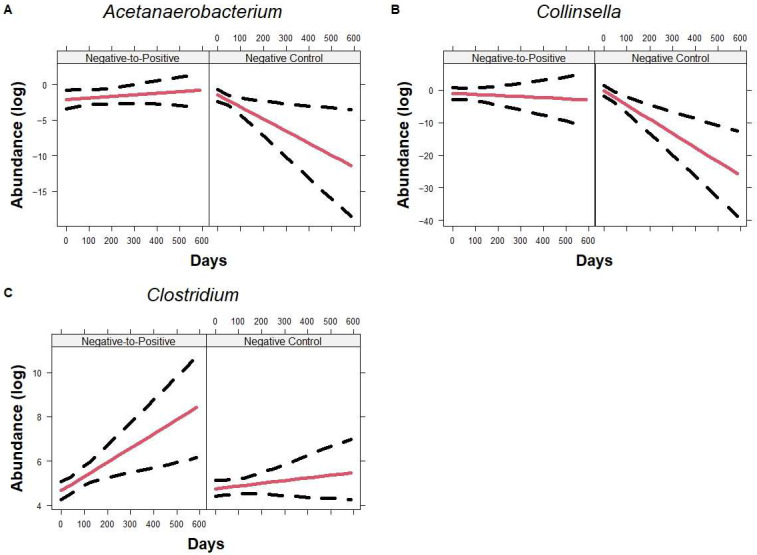
Trajectory of genus using all time points. Line plots showing genera *Acetanaerobacterium* (**A**) and *Collinsella* (**B**), and *Clostridium* (**C**) changes over time in Negative-to-Positive group and Negative Control group. Lines represent the fitted model and the dotted line represents the 95% CIs. Data are presented in natural log scale.

At the genus level, when compared to the Negative Control group, *Faecalitalea* (*p* = 0.0091), *Family XIII AD3011 group* (*p* < 0.0001), *Lactococcus* (*p* = 0.031), *Negativibacillus* (*p* = 0.011), *Ruminococcaceae NK4A214 group* (*p* = 0.010), *Ruminococcaceae UCG-002* (*p* = 0.0010), *Ruminococcaceae UCG-004* (*p* = 0.011), and *Subdoligranulum* (*p* = 0.032) decreased significantly over time in Negative-to-Positive patients (Figure 7). Additionally, *Faecalitalea* (*p* = 0.0057), *Family XIII AD3011 group* (*p* = 0.020), *Ruminococcaceae UCG-002* (*p* < 0.001), and *Ruminococcaceae UCG-004* (*p* = 0.011) were significantly higher at the baseline in the Negative-to-Positive group. In contrast, *Acetanaerobacterium* and *Collinsella* (Figure 8A or Figure 8B) did not change over time in the Negative-to-Positive group but decreased significantly in the Negative Control group, *p* = 0.0046 and *p* = 0.0032, respectively. *Clostridium* (Figure 8C) increased significantly in the Negative-to-Positive group but not in the Negative Control group, *p* = 0.035.

**Figure 9:**
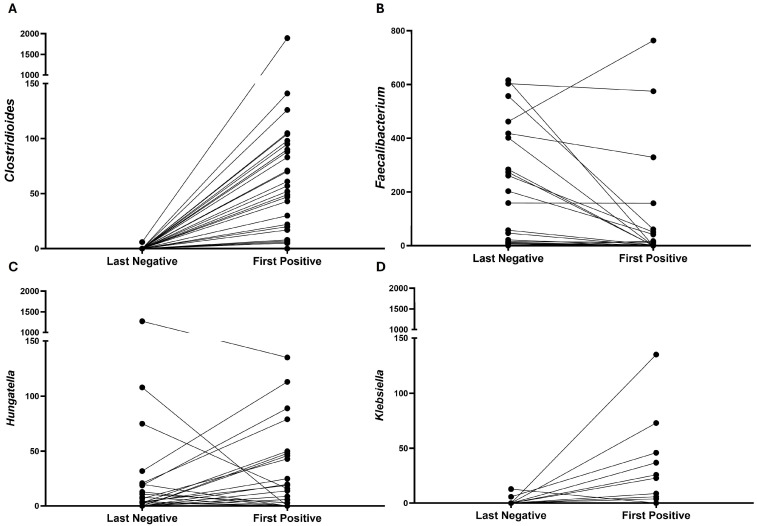
Changes of genus in Negative-to-Positive group. Line plots showing the changes from last negative samples to first positive samples in genus *Clostridioides* (**A**), *Faecalibacterium* (**B**), *Hungatella* (**C**), and *Klebsiella* (**D**).

In Negative-to-Positive patients, the family *Enterobacteriaceae* increased significantly (Table 2, Figure 5B) in the first positive samples compared to the last negative samples, *p* = 0.0094. Additionally, the genera *Clostridioides*, *Hungatella*, and *Klebsiella* had significantly higher levels in the first positive samples while *Faecalibacterium* was much higher before transitioning (Table 2, Figure 9).

## 4. Discussion

In this study, we characterized microbiome changes in patients who transitioned from *C. difficile* negative to *C. difficile* positive using systematic stool surveillance tests. Although no differences were observed in the Shannon index between the groups, we demonstrated significant differences in microbiome composition between last negative and first positive samples in Negative-to-Positive patients. Moreover, we also identified longitudinal compositional differences between groups as well as before and after transitioning to *C. difficile* positive in Negative-to-Positive patients. Collectively, these findings suggest that patients that transitioned from *C. difficile* negative to *C. difficile* positive might exhibit distinct gut microbiome changes compared to healthy individuals or those colonized with *C. difficile*.

Among various risk factors associated with CDI that can lead to disruption of microbiota, antibiotic use is the most common one. A recent study using longitudinal stool samples showed that antibiotic treatment disrupts the gut microbiome, leading to changes in the composition and function of specific microbes [7]. In the current study, the enrolled patients had a median age of 65 years and the majority (94%) had exposure to antimicrobial medications, which made them susceptible to CDI or microbiota dysbiosis.

Decreases in the Shannon index were observed for all three groups in the first 3 weeks and the results were similar after accounting for antibiotic use [19]. Positive Control patients showed a steeper slope, although it was not significantly different from the other groups. The reduction in the Shannon index suggests the underlying mechanism might be more complicated than just being infected with CDI alone. Our study found a significant difference in beta diversity, which was consistent with a study conducted in CDI patients and non-CDI subjects with an average age over 60 years, where Stewart et al. [20] reported significant differences in both bacterial and fungal community composition between these two groups. These data suggest that distinct microbial community structures may exist after *C. difficile* infection, although patients’ conditions in hospital might also play a role.

The first positive samples in Negative-to-Positive patients had less Bacteroidota and Bacillota but more Pseudomonadota compared to the last negative samples, which were consistent with previous findings [21,22,23,24]. The *Lachnospiraceae*, *Oscillospiraceae*, and *Bacteroidaceae* families dominate the communities observed in the gut of healthy individuals [25]. Of which, *Lachnospiraceae* and *Oscillospiraceae* were from the Bacillota phylum. We did not find significant differences in Bacillota between the two groups despite the drop in both Positive Control and Negative-to-Positive patients in the first 3 weeks. Of note, the decrease in Bacillota in the Negative Control group was not as obvious as in the other two groups while Actinomycetota dropped in the Positive Control group significantly compared to the other two groups. *Lachnospiraceae* increased significantly over time in the Negative-to-Positive group compared to the Negative Control group, which was similar to some studies reported previously [26,27,28] but in the opposite direction to others [29,30,31]. We also found that genera *Ruminococcaceae UCG-002*, *Ruminococcaceae UCG-004*, *Ruminococcaceae NK4A214 group*, and *Subdoligranulum* from the *Oscillospiraceae* family and Bacillota phylum decreased significantly in the Negative-to-Positive group compared to the Negative Control group, although previous studies suggested mixed findings [27,29,30,32,33]. We also saw a reduction in *Faecalibacterium*, from the *Oscillospiraceae* family and the Bacillota phylum, in the Negative-to-Positive group over time. Although the decrease over time was not significantly different from the Negative Control group, *Faecalibacterium* was much higher before transitioning as reported previously [20,29,30,34,35,36]. Certain Pseudomonadota such as the family *Enterobacteriaceae* increased in the first positive samples after the transition and similar results have been reported in studies comparing healthy adults with CDI and non-CDI patients with diarrhea [26,29]. The *Enterobacteriaceae* family includes *Escherichia coli* and other potentially pathogenic bacteria which pose public health concerns [37]. Our data show that the genus *Klebsiella* from the *Enterobacteriaceae* family increased significantly after transition to *C. difficile* positive compared to the last negative samples as shown previously [30,31,33,35,36]. Actinomycetota, which may be beneficial in improving gut microbiome health [38], dropped significantly in Positive Control patients but not in the other two groups in the first 3 weeks. The trajectories did not differ in Negative-to-Positive and Negative Control groups in the long term. These data suggest that the decreases in members from beneficial species, accompanied by the drops in members of harmful species, may be indicative of the dysbiosis of the gut microbiome in patients who transitioned from *C. difficile* negative to positive and those who always tested positive.

This study has several limitations. First, this is a retrospective cohort study where some information such as detailed antibiotic exposures (administration, duration of use, and discontinuation) and time point of weight loss cannot be obtained. Second, the current study exclusively detects toxigenic *C. difficile* strains. Therefore, the transitions involving non-toxigenic strains and hypervirulent strains were not captured. Additionally, a CDI diagnosis in each group was not obtained. Third, *C. difficile* can exist as inactive spores transiting the gut or as a true colonization and a positive qPCR cannot conclusively differentiate between these two conditions. Therefore, our findings only reflect the microbiome structural changes due to the presence of *C. difficile*. Fourth, long-term trajectories of Positive Control patients cannot be studied as we only had 18 Positive Control patients with a much shorter follow-up time and fewer numbers of stool samples. Samples in the Negative-to-Positive and the Negative Control groups became sparse and variabilities increased dramatically at later time points, especially for the patients that transitioned from negative to positive *C. difficile*. Fifth, metabolomics data would be useful to see how functions in the gut microbiota might be affected. Metabolic activities of the gut microbiota, such as short-chain fatty acid (SCFA) production, might be altered. It will be interesting to see how metabolome changes and integrated analysis of metabolome and microbiome data may be informative. Various metabolites produced by a human host and gut microbiota may interact with *C. difficile*.

## 5. Conclusions

In summary, we found significant differences in the microbiome composition before and after the transition and longitudinal taxon count changes over time in Negative-to-Positive patients. These results provide characterizations of baseline and longitudinal microbiome compositional changes in patients who transitioned from *C. difficile* negative to *C. difficile* positive with a new insight in CDI progression. Moreover, these changes play an important role in both the emergence and intensification of gut microbiome dysbiosis in patients who transitioned from *C. difficile* negative to positive and those who always tested positive. Intervention that preserves the protective species such as members of the Bacillota phylum from decreasing in these patients before transitioning may be beneficial. Further studies are needed to prospectively examine the structural microbiome changes over time in these patients to gain a more complete understanding and establish a causal relationship.

## Figures and Tables

**Table 1:** Patients’ characteristics by groups.

	Total	Negative to Positive (*n* = 42)	Negative Control (*n* = 47)	Positive Control (*n* = 18)	*p* Value
Gender					
Male	59 (55)	22 (52)	26 (55)	11 (61)	0.82
Age	65 (54, 71)	63 (51, 73)	65 (55, 70)	66 (54, 72)	0.12
Race					0.019
White	87 (82)	33 (80)	43 (91)	11 (61)	
AA	15 (14)	5 (12)	4 (9)	6 (33)	
Other	4 (4)	3 (7)	0 (0)	1 (6)	
ICU stay	19 (18)	6 (14)	13 (28)	0 (0)	0.024
Weight loss	40 (37)	12 (29)	22 (47)	6 (33)	0.19
Hem-onc	99 (93)	38 (90)	45 (96)	16 (89)	0.49
BMT	49 (46)	18 (43)	25 (53)	6 (33)	0.31
Comorbidities * (n)	6 (4, 9)	6 (3, 8)	6 (4, 10)	6 (3, 9)	0.55
CHF	15 (14)	3 (7)	10 (21)	2 (11)	0.17
Antibiotics	101 (94)	41 (98)	46 (98)	14 (78)	0.010
Bactrim	33 (31)	13 (31)	18 (38)	2 (11)	0.10
Amoxicillin **	14 (13)	2 (5)	12 (26)	0 (0)	<0.01
IV vancomycin	47 (44)	19 (45)	26 (55)	2 (11)	<0.01
PO vancomycin	15 (14)	12 (29)	1 (2)	2 (11)	<0.001
Cefepime	61 (57)	25 (60)	29 (62)	7 (39)	0.23
Cefazolin	5 (5)	4 (10)	1 (2)	0 (0)	0.19
Ceftriaxone	14 (13)	4 (10)	7 (15)	3 (17)	0.65
Azithromycin	17 (16)	3 (7)	11 (23)	3 (17)	0.12
PO cephalosporin	5 (5)	3 (7)	1 (2)	1 (6)	0.48
Linezolid	12 (11)	3 (7)	8 (17)	1 (6)	0.33
Dapsone	8 (7)	3 (7)	5 (11)	0 (0)	0.44
Doxycycline	8 (7)	2 (5)	6 (13)	0 (0)	0.19
Aztreonam	5 (5)	2 (5)	2 (4)	1 (6)	>0.99
Macrolide	1 (1)	1 (2)	0 (0)	0 (0)	0.56
Daptomycin	9 (8)	0 (0)	9 (19)	0 (0)	<0.01
Cefoxitin	1 (1)	0 (0)	1 (2)	0 (0)	>0.99
Metronidazole	16 (15)	7 (17)	9 (19)	0 (0)	0.12
Quinolones	64 (60)	21 (50)	37 (79)	6 (33)	<0.001
Clindamycin	1 (1)	1 (2)	0 (0)	0 (0)	0.56
Carbapenems	16 (15)	3 (7)	12 (26)	1 (6)	0.029
Zerbaxa	0 (0)	0 (0)	0 (0)	0 (0)	N/A
Piptazo	40 (37)	10 (24)	26 (55)	4 (22)	<0.01
Pen VK	5 (5)	0 (0)	5 (11)	0 (0)	0.040
Antibiotics with activity against anaerobes	88 (82)	33 (79)	45 (96)	10 (56)	<0.001
Other	6 (6)	0 (0)	6 (13)	0 (0)	0.021
Antifungals	79 (74)	28 (67)	41 (87)	10 (56)	0.011
Azole	76 (71)	27 (64)	39 (83)	10 (56)	0.043
Echinocandin	17 (16)	3 (7)	14 (30)	0 (0)	<0.01
Amphotericin B	2 (2)	0 (0)	2 (4)	0 (0)	0.65

Data were presented as *n* (%) or median (IQR); ICU: intensive care unit; Hem-onc: hematology oncology; BMT: bone marrow transplant; CHF: congestive heart failure; N/A: not applicable; * Based on ICD (International Classification of Diseases) Codes; ** Amoxicillin or amoxicillin/clavulanate or ampicillin/sulbactam.

**Table 2:** Comparison of abundance of last negative vs. first positive samples in Negative-to-Positive patients.

	Last Negative (*n* = 42)	First Positive (*n* = 42)	*p* Value
Phylum			
Pseudomonadota	76 (4, 271)	134 (54, 1164)	0.0075
Family			
*Enterobacteriaceae*	5 (0, 191)	54 (0, 902)	0.0094
Genus			
*Clostridioides*	0 (0, 0)	21 (0, 83)	0.0065
*Faecalibacterium*	0 (0, 159)	0 (0, 5)	0.0027
*Hungatella*	0 (0, 8)	0 (0, 43)	0.048
*Klebsiella*	0 (0, 0)	0 (0, 4)	0.0049

Data were presented as median (IQR).

## Data Availability

The original contributions presented in this study are included in the article/Supplementary Material. Further inquiries can be directed to the corresponding author.

## Supplementary Materials

The following supporting information can be downloaded at: https://www.mdpi.com/article/10.3390/microorganisms13102277/s1, Supplementary Figure S1: Bacterial composition at the phylum level among groups over time. Stacked bar plots showing changes in the relative abundance of the most abundant phyla by month in Negative-to-Positive group (A), Negative Control group (B), and Positive Control group (panel C). Supplementary Figure S2: Trajectory of Phyla using all time points. Line plots showing phyla Actinomycetota (A), Bacteroidota (B), Bacillota (C), Pseudomonadota (D), and Verrucomicrobiota (E) changes over time in Negative-to-Positive group and Negative Control group.
