# Supplementary figures and images for: Longitudinal Gut Microbiome Changes Associated with Transitions from C. difficile Negative to C. difficile Positive on Surveillance Tests
Source: Microorganisms. 2025 Sep 29;13(10):2277. doi: 10.3390/microorganisms13102277 (PMC12566496; doi:10.3390/microorganisms13102277)

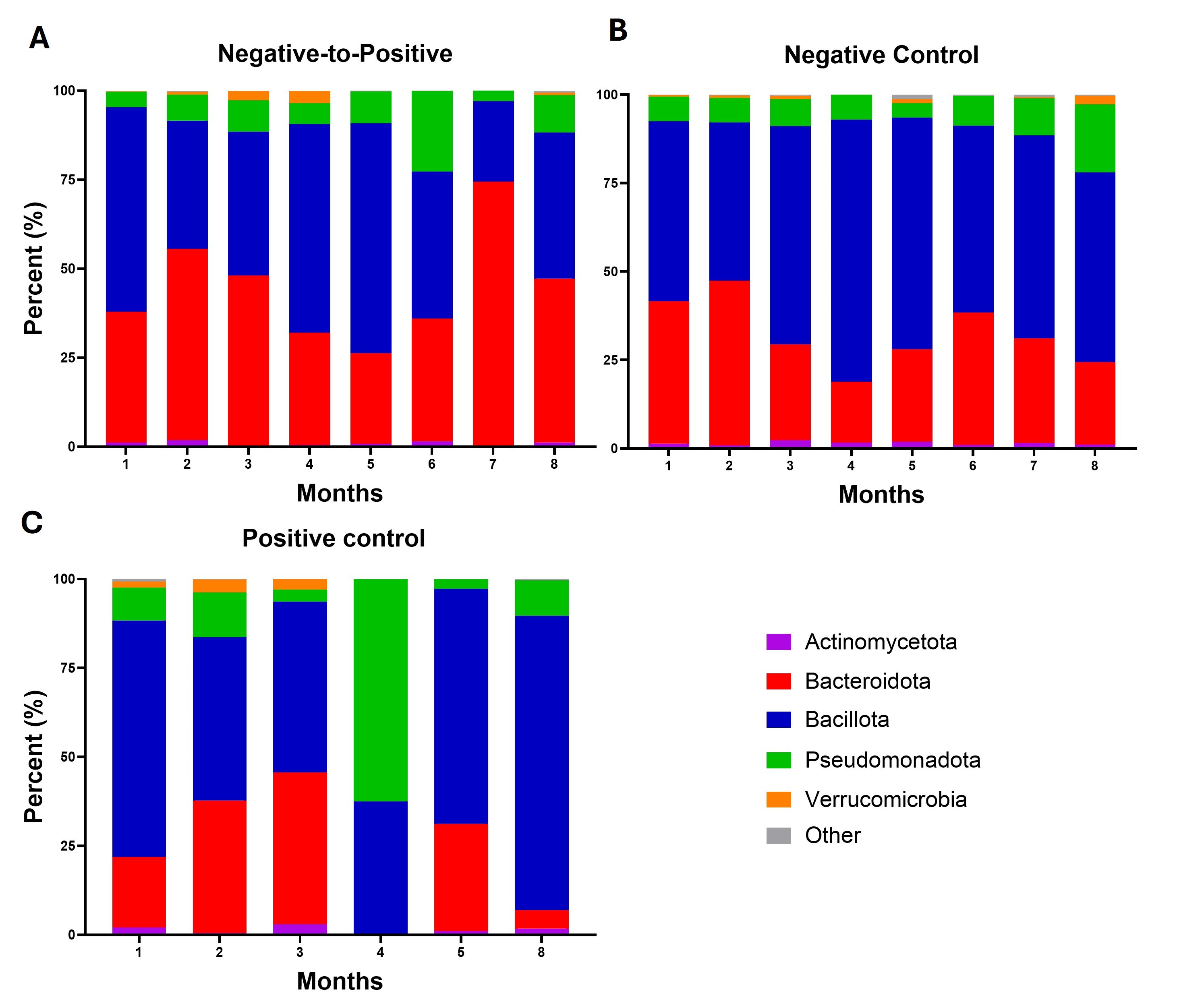

Supplement: Supplementary file 1 [file microorganisms-13-02277-s001.zip › Supplementary Figure 1.jpeg]

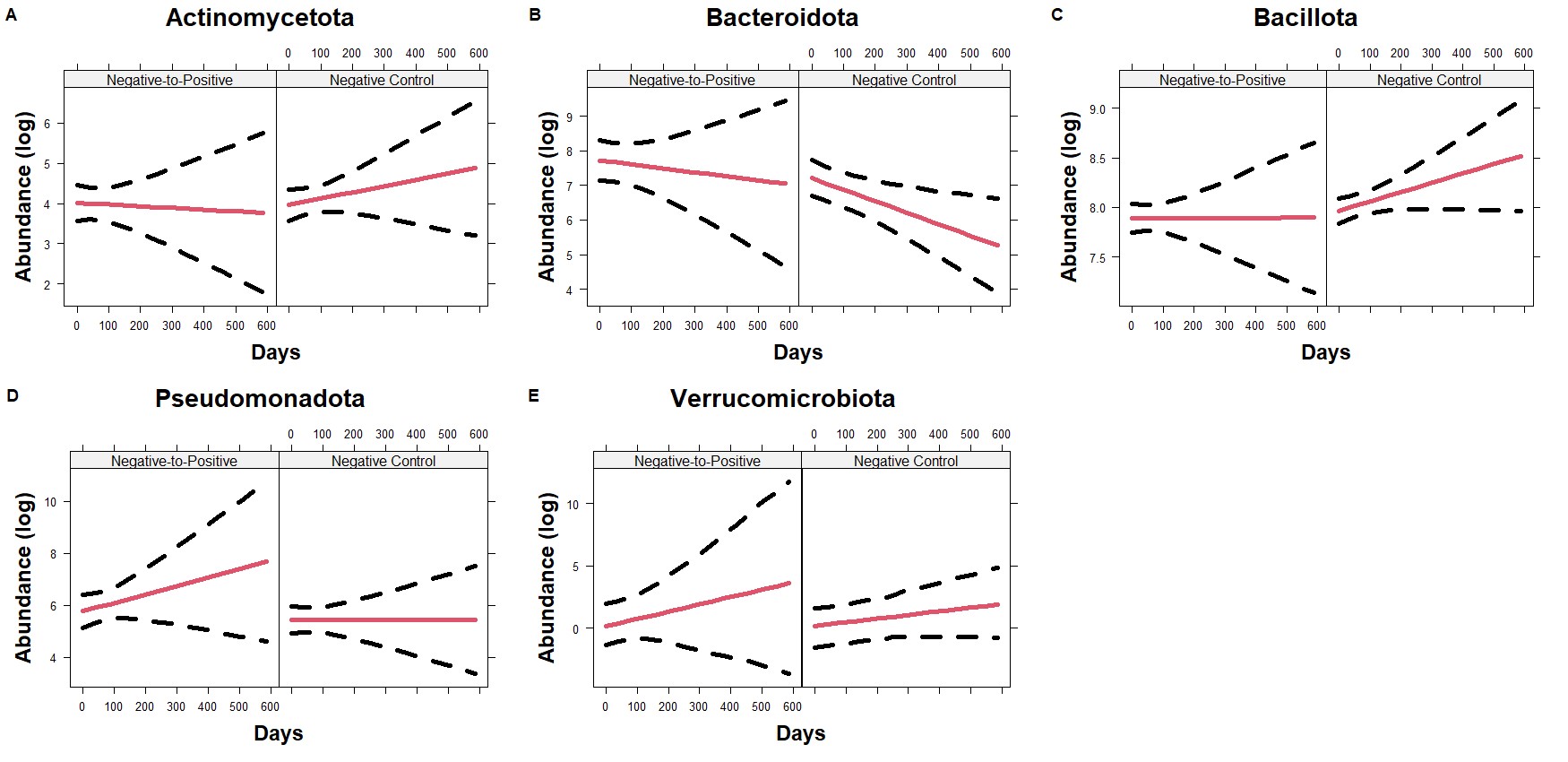

Supplement: Supplementary file 1 [file microorganisms-13-02277-s001.zip › Supplementary Figure 2.jpeg]
